# The Identification of Congeners and Aliens by *Drosophila* Larvae

**DOI:** 10.1371/journal.pone.0136363

**Published:** 2015-08-27

**Authors:** Francisco Del Pino, Claudia Jara, Luis Pino, María Cristina Medina-Muñoz, Eduardo Alvarez, Raúl Godoy-Herrera

**Affiliations:** 1 Laboratorio de Etología, Genética y Evolución de la Conducta, Programa de Genética Humana, Instituto de Ciencias Biomédicas, Facultad de Medicina, Universidad de Chile, Independencia 1027, Santiago, Chile; 2 Departamento de Biología, Facultad de Ciencias, Universidad de Playa Ancha de Ciencias de la Educación, Valparaíso, Chile; Alexander Fleming Biomedical Sciences Research Center, GREECE

## Abstract

We investigated the role of *Drosophila* larva olfactory system in identification of congeners and aliens. We discuss the importance of these activities in larva navigation across substrates, and the implications for allocation of space and food among species of similar ecologies. Wild type larvae of cosmopolitan *D*. *melanogaster* and endemic *D*. *pavani*, which cohabit the same breeding sites, used species-specific volatiles to identify conspecifics and aliens moving toward larvae of their species. *D*. *gaucha* larvae, a sibling species of *D*. *pavani* that is ecologically isolated from *D*. *melanogaster*, did not respond to *melanogaster* odor cues. Similar to *D*. *pavani* larvae, the navigation of *pavani* female x *gaucha* male hybrids was influenced by conspecific and alien odors, whereas *gaucha* female x *pavani* male hybrid larvae exhibited behavior similar to the *D*. *gaucha* parent. The two sibling species exhibited substantial evolutionary divergence in processing the odor inputs necessary to identify conspecifics. *Orco* (*Or83b*) mutant larvae of *D*. *melanogaster*, which exhibit a loss of sense of smell, did not distinguish conspecific from alien larvae, instead moving across the substrate. *Syn* ^*97CS*^ and *rut* larvae of *D*. *melanogaster*, which are unable to learn but can smell, moved across the substrate as well. The *Orco* (*Or83b*), *Syn* ^*97CS*^ and *rut* loci are necessary to orient navigation by *D*. *melanogaster* larvae. Individuals of the Trana strain of *D*. *melanogaster* did not respond to conspecific and alien larval volatiles and therefore navigated randomly across the substrate. By contrast, larvae of the Til-Til strain used larval volatiles to orient their movement. Natural populations of *D*. *melanogaster* may exhibit differences in identification of conspecific and alien larvae. Larval locomotion was not affected by the volatiles.

## Introduction

The identification of congeners and aliens is an essential capability enabling animals to efficiently use ecological resources [1]. In species as diverse as insects, mice and humans, behaviors linked with recognition of individuals have evolved toward the formation of societies to improve access to ecological resources and increase individual fitness [2, 3]. In *Drosophila*, most studies on identification of individuals have focused on adults due to the importance of recognition for mating and reproduction [4]. How *Drosophila* larvae distinguish and navigate toward congeners merits particular attention because of the significance of these behaviors in the allocation of space and food among species with similar ecologies and their consequences for the expansion and evolution of populations [5–9]. Therefore, studies of species-specific cues that guide and orient the movement of *Drosophila* larvae in the wild are of great importance.

*Drosophila* larvae develop in dynamic and variable environments. Moreover, the gradual desiccation of *Drosophila* breeding sites is a selective pressure that acts on larval behavior [7, 10, 11]. The process of decay in fruit where a number of *Drosophila* species live produces volatiles as alcohols, esters and fatty acids that surround the larvae [12]. The fruit itself, the microorganisms responsible for fermentation and conspecific and alien larvae also emit odors [13, 14]. The proportions of these compounds change over time [15]. To live in these changing environments larvae are equipped with highly sophisticated olfactory and gustatory receptors and brain structures that respond to a variety of stimuli [16, 17], indicating their ecological importance for the larvae. Detailed information is available on the cellular biology, genetics and development of the structure and functioning of the olfactory and gustatory systems of *Drosophila* larva [18]. However, the role of those structurally complex sensorial systems in the ecology of *Drosophila* at breeding sites is unclear because very little is known regarding *Drosophila* larva routines in the wild [19]. Here we examine the role of *Drosophila* larva olfactory system in the identification of congeners and aliens and in navigation across substrates. Our study may contribute to an understanding of neurobiology, genetics, ecology and evolution of *Drosophil+a* larval behavior.

We hypothesized that identification at distance of conspecifics and aliens by *Drosophila* larvae might be based on species-specific volatiles [6]. As these olfactory cues are processed by the nervous system, the larvae would navigate toward congeners, avoiding larvae of other species and grouping in proximity to the conspecific source of volatile emissions. This function might be detected under laboratory conditions in which third-instar larvae searching for pupation sites are incited to distribute themselves in space according larval odors of the same and other species. We conducted this essay under illuminated conditions. Thus, we presumed that larvae used primarily chemosensory cues to explore the environment. To verify this assumption, we tested *Orco* (*Or83b*) mutant larvae of *D*. *melanogaster*, which are unable to smell but can taste and see [17].

We also reasoned that the relatively short duration of the breeding sites caused principally by loss of water [20], could make it essential for the larvae to be able to rapidly scrutinize the fruit. The larvae could save time associating conspecific larval odors with appropriate places to feed and pupate. This behavior could be amalgamated with the detection of alien larval odors. Such odors might indicate locations colonized by other species. We tested these hypotheses by using mutant larvae of *D*. *melanogaster* that are unable to learn but can smell and see (*Syn* ^*97CS*^ and *rut* larvae) [17, 21–26].

We also addressed the larval olfactory responses of the endemic sibling species *Drosophila pavani* and *Drosophila gaucha*. Although larvae of *D*. *melanogaster* and *D*. *pavani* cohabit on the same fruits, the larvae of *D*. *gaucha* are ecologically isolated from these two species [6]. We tested the response of larvae of the two sibling species to *D*. *melanogaster* larval odors. We also investigated the olfactory responses of *D*. *pavani* x *D*. *gaucha* reciprocal hybrids to conspecific and *D*. *melanogaster* larval odors. These studies can provide valuable insight into the olfactory world of *Drosophila*.

## Materials and Methods

### Collection of decaying fruits and larval behavior in the wild

We observed the activities of *Drosophila* larvae on a variety of decaying substrates (grape, apple, pear, peach, prickly pear and cactus cladode tissue (*Opuntia ficus-indica*) in Til-Til, 33°05’00”S, and Trana, 35° 52’ 00” S, Central Valley of Chile. To substantiate that larvae of several species cohabit in the same fruits, we randomly collected decaying fruits (N = 502 fruits), and taxonomically identifying the adults as they emerged from each of the fruits.

### Subjects

We tested wild-type larvae of natural Chilean populations (Til-Til and Trana strains) and laboratory stocks (Oregon R-c and Canton–Special strains) of *D*. *melanogaster*. We also examined larvae of the *vestigial* (*vg*) strain. The *vg* strain and the Oregon R-c strain differ in certain larval behaviors. For example, Oregon R-c larvae dig deeper into the substratum than *vg* larvae [27]. We also tested three neurological mutants derived from the Canton-Special (CS) strain of *D*. *melanogaster*. We reasoned that the olfactory-mediated behaviors require normal olfactory receptor functioning. The larval perception of odorants in the *Orco* (*Or83b*) mutant strain is blocked because the dendritic localization of the receptors is lost [17, 21]. Thus, the *Orco* mutation disrupts behavioral and electrophysiological responses to many odorants [17]. To obtain clues regarding social larval odor-based learning, we tested the *Syn* ^*97CS*^ and *rut* learning mutant larvae. The *Syn* ^*97CS*^ mutation affects presynaptic vesicle release in the entire larval brain, and olfactory associative learning is reduced in approximately 50% of these larvae compared with the CS larvae; however, the responsiveness to stimuli and motor performance in untrained animals are normal [22–24]. The *rut* locus participates in olfactory conditioning learning in *D*. *melanogaster*, and it is expressed in the neurons located in the larval and adult mushroom bodies; *rut* does not affect larval locomotion or responsiveness to stimuli [25, 26].

The wild type Trana strain of *D*. *melanogaster* was established with adults that emerged from grape (*Vitis vinifera*, País Variety); no other *Drosophila* species emerged from the decaying fruit. The wild-type Til-Til strain of *D*. *melanogaster* was formed with adults that emerged from decaying prickly pear fruits collected. The Til–Til and Trana larvae used in the laboratory experiments were fourth-generation.

We are indebted Don Rodrigo Pica owner of the Fundo Trana in Cauquenes, VIII Region of Chile, who kindly issued the permission for our field studies in that land. We are also grateful to Don Juan Ignacio Herrera and Don Gonzalo Herrera owners of the Fundo La Capilla in Til-Til who issued the permission to collect flies and fruits, and for their tolerance while we invaded their land. We thank Dr Bertram Gerber, University of Würzburg in Germany. He sent us the Canton-Special (CS), *Orco* (*Or83b*), *Syn* ^*97CS*^ and *rut* strains to our laboratory.

We also investigated the effect of larval olfactory cues on navigation of *D*. *pavani* (La Florida strain, 33° 33’ 00”S), its sibling *D*. *gaucha* (Buenos Aires strain, 34°20’00”S) and the F_1_ reciprocal hybrid larvae. The emergency of adults of *D*. *melanogaster* and *D*. *pavani* from the same decaying fruit unit in the wild, suggested that *D*. *pavani* larval odors play a role in the orientation of the movement of *D*. *melanogaster* larvae. These two endemic South American sibling species belong to Subgenus *Drosophila*, *mesophragmatica* group [28–30]. *D*. *pavani* is predominantly Andean in distribution, whereas *D*. *gaucha* is distributed in Argentina, Uruguay and southern Brazil [29]. Under laboratory conditions, the two species can produce viable but sterile hybrids [31]. *D*. *pavani* and *D*. *gaucha* have similar development durations of molting, wandering and pupating [32].

The strains were maintained by mass culture at 24 ± 1°C 70% (*D*. *melanogaster*) and 18 ± 1°C, 80% humidity (*D*. *pavani* and *D*. *gaucha*). *D*. *pavani* and *D*. *gaucha* grow better at this temperature and humidity than at 24°C. All stocks were maintained under constant light because the laboratory was not equipped to change the light/dark period.

### Crosses

Fifteen-day-old *D*. *pavani* (La Florida strain) and *D*. *gaucha* (Buenos Aires strain) males and females were reciprocally crossed. At this post-emergence age, individuals are sexually competent [32]. Homogametic mating within strains served as controls for the interspecific crosses. Crosses between the La Florida (*D*. *pavani*) and Buenos Aires (*D*. *gaucha*) strains provided abundant hybrid larvae of both sexes [31, 32].

### Larva collection

Groups of 40–50 inseminated females of *D*. *melanogaster*, *D*. *pavani*, *D*. *gaucha*, and *D*. *pavani* females and *D*. *gaucha* females crossed with males of the other species were allowed to oviposit for 2–3 h on plastic spoons containing the culture medium [31]. Thirty eggs of the species, strains and hybrids were randomly collected with a dissecting needle. Each batch of eggs was incubated on fresh spoons for 96–100 h at 24°C (*D*. *melanogaster* strains) and for 168–172 h at 18°C (*D*. *pavani*, *D*. *gaucha* and the hybrids).

One hour before an experiment, third-instar larvae were collected from the glass wall of rearing bottles, washed twice with distilled water, and identified by the presence of protruded anterior spiracles [33]. All larvae were raised in half-pint bottles at 24°C (*D*. *melanogaster*) and 18°C (*D*. *pavani* and *D*. *gaucha*) on Burdick’s medium [34].

### Treatments

We used two 2 x 2 cm pieces of Whatman cellulose filter papers. In the first treatment, one of the papers was moistened in sterile Burdick’s medium, whereas the other was moistened in Burdick’s medium used for 4–5 days by Oregon R-c larvae of *D*. *melanogaster* (or *D*. *pavani*) larvae. Before transfer to Petri dishes, the two filter paper types were carefully examined under stereomicroscope to verify that no food had adhered to the surface. In the second treatment, one of the papers was moistened in food used by larvae of the strain (Canton-Special, Til-Til, Trana, *vestigial*, *Orco* (*Or83b*), *Syn* ^*97CS*^ and *rut* strains of *D*. *melanogaster*) and the other filter paper was moistened in food processed by the Oregon R-c larvae. The test for Oregon R-c larvae was an Oregon R-c filter paper, and a Canton-Special filter paper. In the third treatment, we used one filter paper moistened in food occupied by larvae of *D*. *melanogaster* strains, another moistened in food used by *D*. *pavani* larvae. These treatments were also applied to *D*. *pavani*, *D*. *gaucha* and the interspecific reciprocal hybrid larvae.

For each treatment, 10-cm Petri dishes were filled with 10 ml of 3% agar gel. The two different filter papers were deposited on opposite sides (6 cm of separation) of the agar in Petri dish. Batches of 20 third-instar larvae of each species and strain were introduced into the Petri dishes and gently deposited onto the middle of the agar, 3 cm from each piece of paper. Once the larvae actively moving, we recorded the observed number of larvae on each type of filter paper every 2 min for 20 min. Some larvae only approached the papers. Larvae detected on agar within 1 cm of the border of each paper were counted as belonging to that paper. Replicate measurements (10 replicates, N = 200 larvae) were performed for each strain. To decrease possibility that differences in illumination conditions would interfere with orientation of the larvae in each treatment, we recorded every 5 min the amount of light in four opposite locations near the edge of each of the Petri dishes. The possibility that the substances present in the papers could diffuse through the agar was addressed by testing *Orco* mutant larvae. This mutation does not affect gustatory neurons [17].

### Larva locomotion

We tested four treatments to measure locomotion of the larvae on agar: (i) no odor, except the aroma of agar, (controls); (ii) the aroma of Burdick’s medium; (iii) conspecific larval odors; and (iv) *D*. *pavani* (or *D*. *gaucha* and hybrid) larval cues. For the treatments (ii), (iii) and (iv), a 2 x 2 cm piece of paper impregnated in Burdick’s medium (or the corresponding strains of *D*. *melanogaster*, *D*. *pavani*, *D*. *gaucha* or hybrids) was deposited onto the center of a Petri dish filled with 3% agar gel for 1 hour. The paper was subsequently removed, and a third-instar larva of the strains and species indicated above was gently deposited on the Petri dish agar (N = 50 per strains and species and hybrids). Once the larva started to actively move, locomotion was recorded for 2 min. Locomotion was measured as the number of waves of segmental contraction per minute passing in series along the body [31]. A new Petri dish was used for each larva tested.

### Statistical analysis

#### Larval orientation

We applied a generalized linear mixed model (GLZM) to analyze number of larvae on the filter papers. The explicatory variables (strains, treatments, observation times and their interactions) were treated as categorical variables and fixed factors. For this purpose we used the Poisson distribution. The link function was a log-linear model [35]:

NO OF LARVA LOCATION=STRAINS(REPLICATES)+TREATMENTS+OBSERVATION TIMES+STRAINS X TREATMENTS+STRAINS X OBSERVATION TIMES+TREATMENTS X OBSERVATION TIMES+STRAINS X TREATMENTS X OBSERVATION TIMES.

Response variable=No of larva location

Explanatory variable1=strains with nested replicates

Explanatory variable2=treatments

Explanatory variable3=observation times

**Fig 1:**
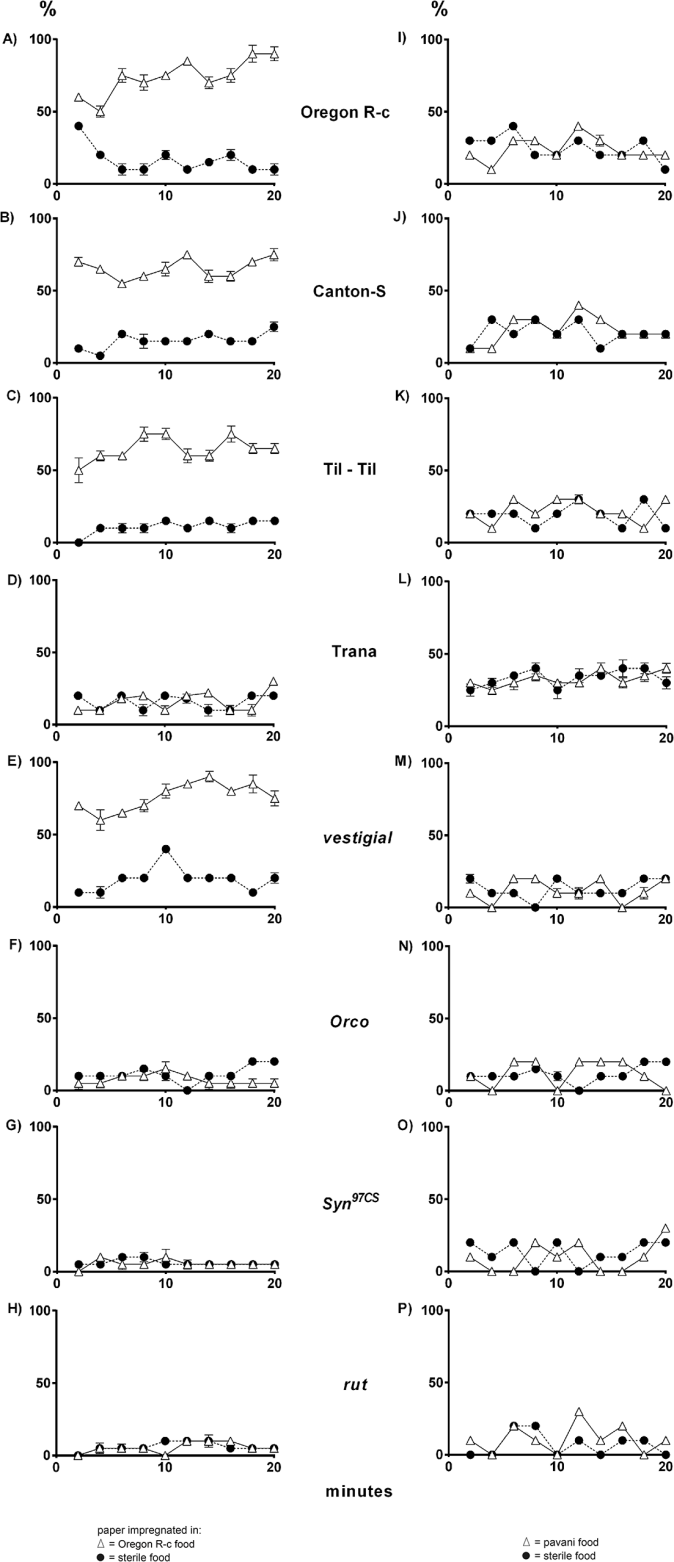
Navigation of third-instar larvae of *D*. *melanogaster* stimulated by conspecific odors and *D*. *pavani* odors. **(A–H),** response to sterile food aroma and food processed by Oregon R-c larvae (*D*. *melanogaster*). **(I–P),** response to sterile food aroma and food worked by La Florida larvae (*D*. *pavani* **).** Navigation toward sterile food, congeners and alien odors is shown as percentage of larvae ± SE arriving at the papers. Black circle (**A–P**), filter paper moistened in sterile food. White triangle (**A–H**), filter paper moistened in food used by Oregon R-c larvae (*D*. *melanogaster*) or La Florida larvae (*D*. *pavani*) (**I–P**). When standard errors are not shown is because they are too small.

In the model, treatments correspond to the two tests conducted with each strain. For example, we compared number of larvae on the papers in the sterile medium/conspecific medium treatment versus the sterile medium/heterospecific medium treatment (for an example see Fig 1**A**–1**H**). We nested replicates within each strain because the number of larvae on the papers could vary randomly across strains of *D*. *melanogaster* and across genotypes within a strain. We also conducted a similar analysis of *D*. *pavani*, *D*. *gaucha* and the hybrid larvae. The Deviance, Pearson **χ** ^2^ and Akaike Information Criterion (AIC) values were also determined to assess how closely the model-based fitted values approximated the observed values [35]. We examined the statistically significant interactions to analyze the effect of each treatment on larval behavior of each strain and species. We applied Wald **χ** ^2^ to assess statistical significance of the explanatory variables [35]; the *G*-test for heterogeneity of the replicates (*D*. *gaucha* and the *gaucha* female x *pavani* male hybrid larvae) was also applied.

#### Larva locomotion analysis

We applied the *t*-test to larval locomotion data from the strains and species examined [35] (N = 50 larvae per strain (species) and treatment).

## Results

### Behavior of *Drosophila* larvae in the breeding sites

**Fig 2:**
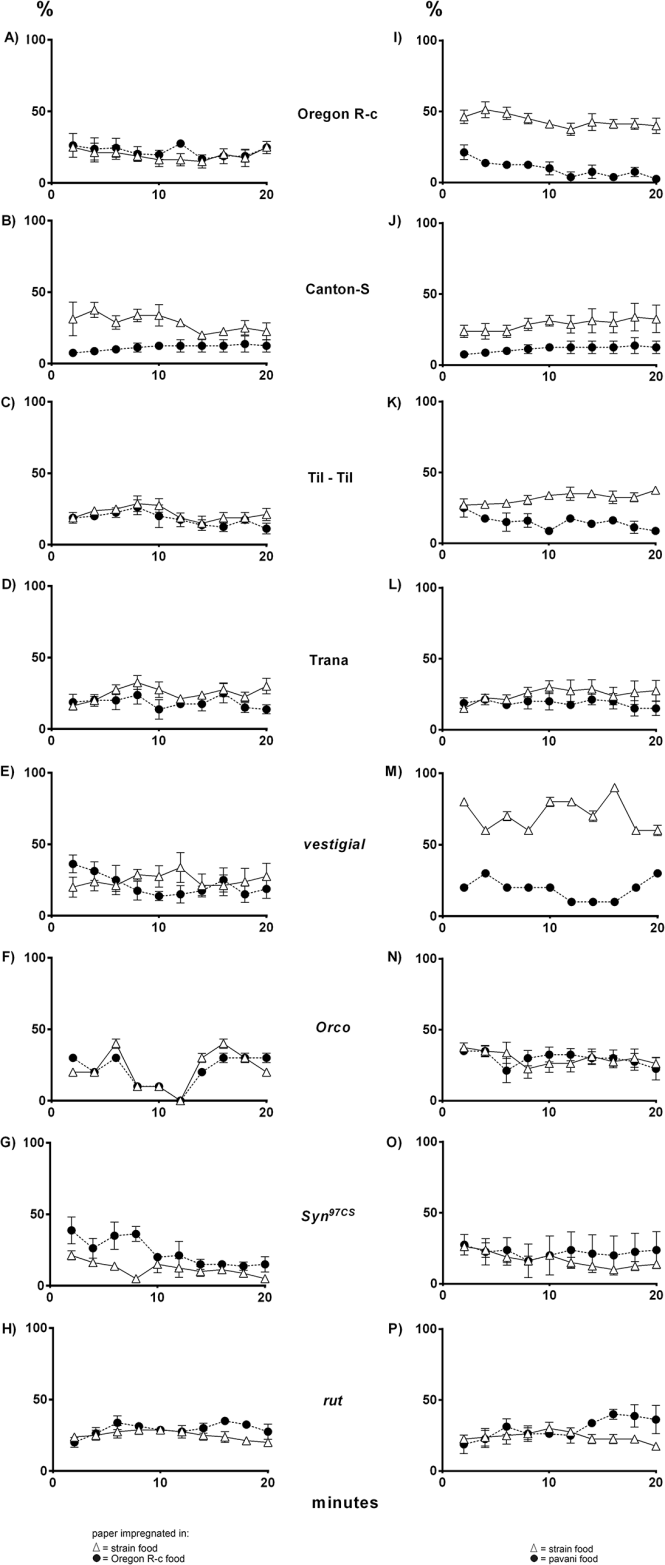
Navigation of *D*. *melanogaster* larvae stimulated by conspecific larval odors of different strains and conspecific and *D*. *pavani* odors. Percentage of larvae on papers impregnated, respectively, in (i) food used by larvae of the strain, white triangles, and (ii) food worked by Oregon R-c larvae, black circles (**A–H**). For Oregon R-c larvae the papers were moistened, respectively, in Oregon R-c food and Canton-Special food. (**I-P**), white triangles, filter paper moistened in food of the strain; black circles, filter paper moistened in *D*. *pavani* food. For further details see Fig 1.

*Drosophila* larvae explore the entire breeding site in the wild, suggesting that they invest a substantial amount of time and energy searching for microorganisms to consume and sites to pupate. To better understand the significance of this behavior, we carefully examined the fruits and tissues. The rearing materials consisted of several connected microhabitats. Some sections of a fruit were in advanced states of decay and dryness, whereas desiccation and fermentation were only beginning in other parts of the same fruit. Other locations in the rearing sites were not fermented (S1 and S2 Figs and Fig 2). These different stages of decay represent changes in ecological conditions that are rapid and difficult to predict. The microhabitats differed also in acidity/alkalinity; acetic acid tended to accumulate in the fruit zones in which decay was advanced. We found *D*. *melanogaster* larvae feeding at pH 3.0; larvae of other species were not observed. Thus, larvae of different *Drosophila* species seem to detect and select chemically different microhabitats within a fruit unit. The desiccation was also heterogeneous. Some parts of a fruit unit were drier than other. At drier sites, we did not find *Drosophila* larvae but instead identified pupae, corroborating the observations of Brncic [19] and Beltramí et al. [14].

### Behavior toward other larvae in the wild

Two or more *Drosophila* species emerged from 79.83% of the collected fruits (N = 502). We identified the sibling *D*. *melanogaster* and *Drosophila simulans*, *Drosophila buzzatii*, *Drosophila hydei*, *Drosophila busckii*, *Drosophila immingrans*, *D*. *pavani*, and *Drosophila nigricruria*. We observed the behavior of larvae toward other individuals. In some instances, navigation was toward other larvae; in other cases, they moved away from other individuals, avoiding contact with them. We conjectured that the larvae were able to identify congeners and aliens at distance, as suggested by Medina-Muñoz and Godoy-Herrera [36]. Some *Drosophila* larvae occasionally touched the spiracles and/or cephalic region of other larvae. Other larvae also scrutinized other individuals by touching them from the cephalic region to the spiracles and/or vice versa. Thus, tactile signals might also be necessary to confirm the identity of congeners and aliens, as in the case of larvae of sibling species.

### Navigation in *D*. *melanogaster* larvae

In the treatment in which one paper was impregnated in sterile food and the other in food processed by Oregon R-c larvae, more than 65% of wild type (the Oregon R-c, Canton-Special and Til–Til strains) and *vg* larvae of *D*. *melanogaster* were detected on the conspecific paper, indicating that when stimulated by conspecific odor cues, the larvae navigated toward them (Fig 1**A–1C** and 1**E**). The response was relatively rapid. At 2–4 min of observation time, approximately 65% of the larva were on or near the conspecific paper; this percentage tended to remain constant over time until the end of the 20 min observation time (Fig 1**A–1C** and 1**E**), suggesting a robust response to conspecific larval odor. In this same assay, approximately 20% of the wild-type Trana larvae were on the sterile food paper and on the paper moistened in food previously exposed to larvae of the same strain; these percentages tended to maintain throughout the entire observation time. The results suggest that Trana larvae did not respond to conspecific odors to navigate across the substrate (Fig 1**D**).

Stimulated by odors emanating from the sterile food paper and Oregon R-c food-impregnated paper, approximately 18% of *Orco* larvae were detected on the papers and 70% on Petri dish agar (Fig 1**F**), suggesting that these larvae did not respond to larval volatiles. In this same essay, approximately 15% of *Syn* ^*97CS*^ and *rut* larvae were detected on each of the papers, and more than 65% moved across agar during the observation time of 20 min (Fig 1**G and** 1**H**). This behavior contrast with that of wild type Canton-Special larvae (Fig 1**B**). We conclude that in *D*. *melanogaster*, *Orco*, *Syn* ^*97CS*^ and *rut* loci are necessary to orient the movement of larvae toward congeners.

Stimulated by the aroma of sterile food and *D*. *pavani* larval odors more than 60% of wild-type (the Oregon R-c, Canton-Special, Til–Til and Trana strains) and *vg* larvae of *D*. *melanogaster* moved on agar; fewer than 15% of the larvae were on each of the papers (Fig 1**I–**1**M**). The percentage of wild type and *vg* larvae on agar tended to remain constant between 2 and 20 min of observation time, suggesting that the larvae identified the odors early (Fig 1**I–**1**M**). Approximately 17% of the *Orco*, *Syn* ^*97CS*^ and *rut* mutant larvae were detected on each of the papers throughout the entire observation time (20 min; Fig 1**N–**1**P**). These findings suggest that volatiles emitted by *pavani* larvae influence navigation of wild-type *D*. *melanogaster* larvae and, again, indicate that the *Orco*, *Syn* ^*97CS*^ and *rut* loci are necessary for identification of congeners and strangers.

In the presence of conspecific odors only, most of the wild type and *vg* larvae of *D*. *melanogaster* moved across the agar, and approximately 15% were recorded on each of the papers (Fig 2**A–**2**E**). Notably, this pattern changed greatly in the presence of congener and *D*. *pavani* odors. The Oregon R-c, Canton-Special, Til-Til and *vg* larvae oriented their movement toward the conspecific paper and approximately 10% of the larvae were on *D*. *pavani* paper (Fig 2**I–**2**K** and 2**M**). Remarkably, Trana larvae again were principally observed on agar in Petri dishes (Figs 1**L**, 1**D**, 2**L** and 2**D**), indicating that these larvae did not respond to congener or *pavani* odors. The *Orco*, *Syn* ^*97CS*^ and *rut* mutant larvae were also principally detected on Petri dish agar (Fig 2**F, 2G, 2H, 2N, 2O** and 2**P**).

### Statistical analysis for *D*. *melanogaster* larval navigation

The ratio of deviance and generalized Pearson χ^2^ calculated values to the degrees of freedom were 1.11 and 1.09, respectively, close to the expected value/df 1.00. We concluded that the model effectively describes the response of larvae of *D*. *melanogaster* to congener and alien larval odors. AIC was 12.35, a relatively small value, suggesting that over-dispersion was absent from data [35].

The calculated Wald χ^2^ values for those explanatory variables were (i) strains, 49.62, *df* = 7, *P* < 0.0001; (ii) treatments, 58.81, *df* = 2, *P* < 0.0001; (iii) strains x treatments, 34.09, *df* = 14, *P* = 0.035. These results show that differences between strains, treatments and interactions between these two variables contribute to variance between and within the strains used. The Wald χ^2^ values for observation times, 7.89, *df* = 9, *P* = 0.996, *NS*; strains x observation times, 10.32, *df* = 63, *P* = 1.00, *NS*); treatments x observation times, 17.36, *df* = 18, *P* = 0.922, *NS*; and strains x treatments x observation times, 21.54, *df* = 126, *P* = 1.0, *NS*, are not statistically important. Based on the significance of the strain x treatment interaction, we also infer strong genotype-by-environment interaction at the level of individual genotypes in the strains of *D*. *melanogaster* tested.

### Navigation of *D*. *pavani*, *D*.*gaucha* and *D*. *pavani* x *D*. *gaucha* reciprocal hybrid larvae

**Fig 3:**
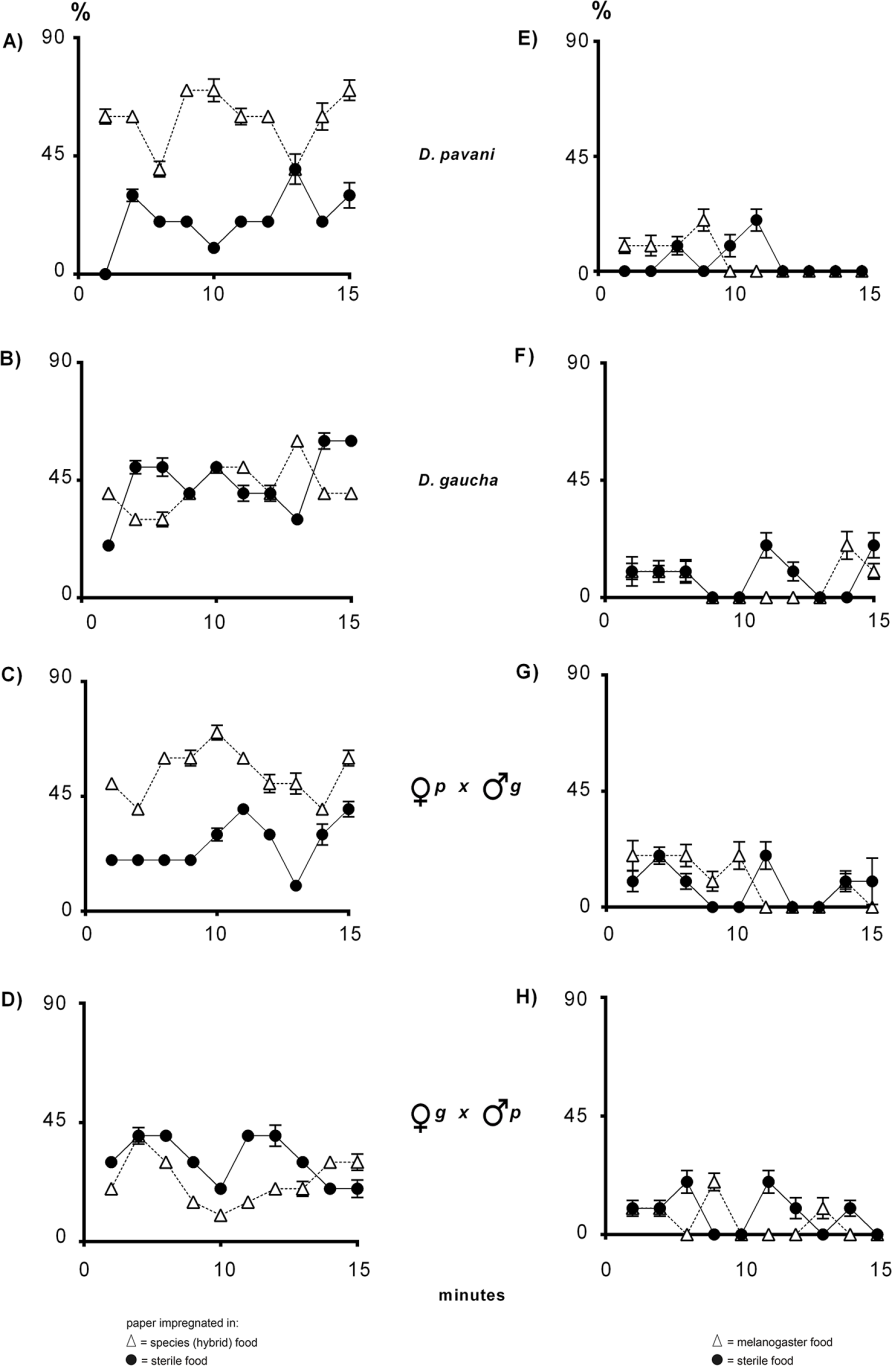
Navigation of *D*. *pavani*, *D*. *gaucha* and hybrid larvae stimulated by conspecific or *D*. *melanogaster* odors. (**A—D**), percentage of larvae on the paper impregnated in sterile food (black circles) and on the paper moistened in food used by larvae of the species or the respective hybrid (white triangles). (**E–H**), percentage of larvae on papers impregnated in sterile food and Oregon R-c (*D*. *melanogaster*) food. See also Fig 1 for further details.

In the sterile food/conspecific food treatment, more than 65% of the *D*. *pavani* and the *pavani* female x *gaucha* male hybrid larvae oriented their movements toward the conspecific paper (Fig 3**A** and 3**C**). Under the same condition, *D*. *gaucha* and the *gaucha* female x *pavani* male hybrid larvae were principally on agar and about a 12% of the larvae were detected on the two papers in the Petri dishes, suggesting that the larvae did not respond to the signals (Fig 3**B** and 3**D**). We assessed these responses by using one paper impregnated in sterile food and the other in *D*. *melanogaster* food. *D*. *pavani* larvae and the *pavani* female x *gaucha* male hybrid larvae moved across the agar in the Petri dish (Fig 3**E** and 3**G**). These results confirm that in *D*. *pavani*, olfaction is required to orient the movement of the larvae. *D*. *gaucha* and the *gaucha* female x *pavani* male hybrid larvae exhibited a similar response to the behavior displayed in the sterile food/conspecific food treatment, suggesting again that they did not use larval volatiles to direct their movements (Fig 3**F** and 3**H**).

**Fig 4:**
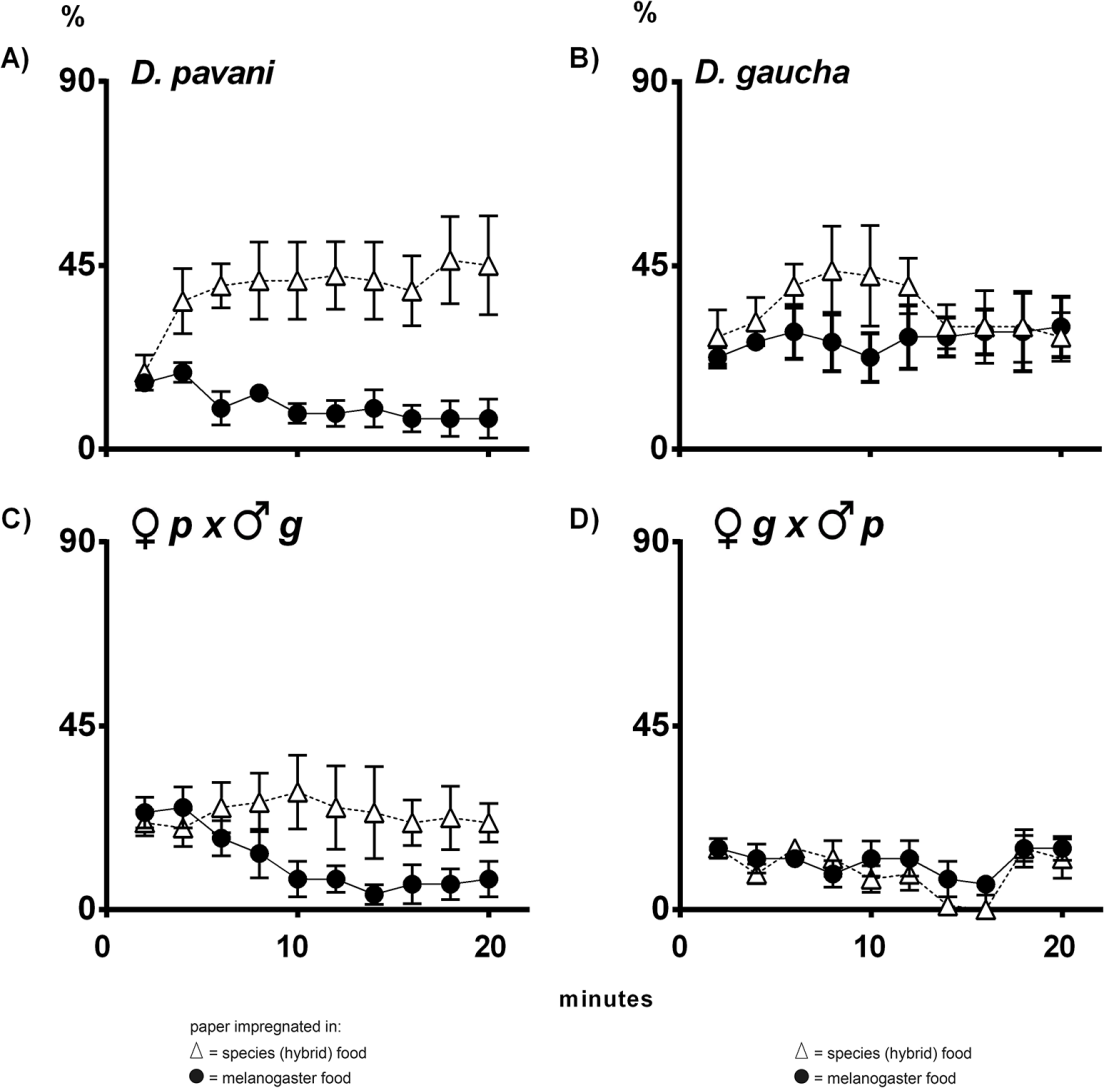
Navigation of *D*. *pavani*, *D*. *gaucha* and the hybrid larvae stimulated at the same time by conspecific and *D*. *melanogaster* odors. White triangles, percentage of larvae on the paper moistened in food used by the species or the respective hybrid. Black circles, percentage of larvae on the paper impregnated in food used by Oregon R-c larvae (*D*. *melanogaster*). (**A**) *D*. *pavani* larvae; (**B**) *D*. *gaucha* larvae; (**C**) *pavani* female x *gaucha* male hybrid larvae; (**D**) *gaucha* female x *pavani* male hybrid larvae.

Stimulated at the same time by food of the species (hybrid) and Oregon R-c food, larvae of *D*. *pavani* and the *pavani* female x *gaucha* male hybrids navigated toward the conspecific paper (Fig 4**A** and 4**C**). By contrast, *D*. *gaucha* and the *gaucha* female x *pavani* male hybrid larvae were on the agar of the Petri dishes (Fig 4**B** and 4**D**). We conclude that in the two sibling species, divergent evolutionary changes associated with the processing of odor volatiles necessary to identify conspecifics and aliens have occurred in the organization and function of the nervous system.

### Statistical analysis for navigation by *D*. *pavani*, *D*. *gaucha* and reciprocal hybrid larvae

The deviance and generalized Pearson χ^2^ values were 108.45, *df* = 27, and 184.08, *df* = 27, respectively, yielding ratio of deviance and Pearson χ^2^ values to degrees of freedom greater than the expected value 1.00. AIC was also high, 228.06. Thus, the binomial GLMM model applied did not fit to the collected data. We examined the four groups of genotypes to identify heterogeneity of the replicates [35]. The *D*. *gaucha* and *D*. *gaucha* female x *D*. *pavani* male hybrid data showed replicate heterogeneity (F_max(3,9)_—test = 7.56, *P* = 0.002), suggesting over-dispersion. Thus, we analyzed *D*. *pavani* and *D*. *pavani* female x *D*. *gaucha* male hybrid data separately from *D*. *gaucha* and *D*. *gaucha* female x *D*. *pavani* male hybrid data.

The GLMM applied to *D*. *pavani* and *D*. *pavani* female x *D*.*gaucha* male data yielded ratios of the deviance and Pearson χ^2^ values to degrees of freedom near the expected value 1.0, suggesting good agreement between the binary model applied to the data and the number of larvae on the conspecific paper. The AIC value was now 21.02, also suggesting a satisfactory goodness of fit between the model and the data [35]. The explanatory variables treatments and species/hybrid x treatments interaction were statistically significant. The calculated Wald χ^2^ values were12.08, *df* = 2, *P* = 0.0070, and 7.93, *df* = 2, *P* = 0.0475, respectively. We did not find statistical significant differences between the behavior of *D*. *pavani* larvae and *D*. *pavani* female x *D*. *gaucha* male hybrid larvae (Wald χ^2^ = 2.35, df = 1, *P* = 0.1253, *NS*).

### Locomotion

Locomotion is essential to move across substrates. We did not observe differences in locomotion between larvae of the corresponding strains and species in the four treatments (**Table 1**). For statistical significance of those differences see **S1 Table** (*t*-test). We conclude that larval volatiles do not modify the function of the neurological circuits that control locomotion at central level.

**Table 1:** Larval locomotion of *D*. *melanogaster* (the Oregon R-c, Canton-Special, Til-Til, Trana, *vestigial*, *Orco*, *Syn* ^*97CS*^ and *rut* strains), *D*.*pavani* (La Florida strain), *D*.*gaucha* (the Buenos Aires strain), and the *pavani* female x *gaucha* male and *gaucha* female x *pavani* male hybrids. Locomotion was measured in four conditions: (i) on agar (agar aroma, controls), (ii) agar plus synthetic food aroma, (iii) on agar plus conspecific odors, and (iv) on agar plus alien food aroma. For *D*. *melanogaster* larvae, *D*. *pavani* food was used as alien food. To *D*. *pavani*, *D*. *gaucha* and the hybrid larvae, alien food means *D*. *melanogaster* (Oregon R-c) food. Locomotion of N = 50 third instar larvae of each strain and species was measured by 2 min (see Materials and Methods).

Species and strains	larval locomotion on agar (controls) (cpm/min)	larval locomotion on agar plus		
		steril food (cpm/min)	conspecific food (cpm/min)	alien food (cpm/min)
***D*. *melanogaster***				
Oregon R-c	108.93 ± 3.27	99.72 ± 8.07	102.92 ± 4.71	103.21 ± 5.03
Canton-Special	96.59 ± 3.86	97.14 ± 4.73	99.62 ± 5.15	97.64 ± 3.92
Til–Til	96.09 ± 7.21	97.69 ± 3.48	96.84 ± 3.82	99.74 ± 3.92
Trana	66.72 ± 6.48	64.35 ± 5.71	70.13 ± 6.19	68.07 ± 5.74
*vestigial*	112.33 ± 5.49	110.85 ± 6.29	109.22 ± 4.73	110.41 ± 3.67
*Orco*	94.61 ± 5.43	96.71 ± 5.03	97.19 + 4.37	95.45 ± 4.76
*Syn* ^*97CS*^	107.96 ± 4.22	99.53 ± 6.46	101.05 ± 2.18	107.48 ± 5.72
*rut*	98.86 ± 5.41	99.06 + 5.79	97.42 ± 6.73	99.76 ± 3.25
***D*. *pavani***	60.31 ± 4.79	61.75 ± 3.25	64.32 ± 4.71	63.93 ± 4.21
***D*. *gaucha***	57.48 ± 5.81	58.12 ± 8.19	56.76 ± 3.15	58.35 ±6.05
**The F**_**1**_ **hybrids**				
*pavani* x *gaucha*	59.58 ± 9.61	55.63 ± 7.46	60.21 ± 5.34	56.31 ± 4.76
*gaucha* x *pavani*	62.79 ± 8.72	65.28 ± 6.39	64.07 ± 7.15	60.32 ± 5.39

## Discussion

In this study we asked where do *Drosophila* larvae go? The absence of information on the behavior of larvae in the wild and on techniques for studying their behavior in the field are partially responsible for the general ignorance of distribution of larvae at the breeding sites, although Godoy-Herrera previously highlighted the potential importance of larval digging behavior in the use of space [27]. We hypothesized that larvae identify other larvae at distance approaching congeners and avoiding aliens. To facilitate this behavior, *Drosophila* larvae would emit species-specific volatiles.

Our study confirms that *D*. *melanogaster* and *D*. *pavani* larvae have the ability to identify other larvae at a distance, orienting their movements toward congeners while avoiding and moving away from aliens. The mechanism of this recognition is the emission of species-specific volatiles (Figs 1 and 2). Del Pino et al. [6] reported that the selection of pupation sites in *D*. *melanogaster* is also significantly affected by *Orco*, suggesting the importance of this locus in the allocation of space and habitat selection. Other olfactory mutations such as *poxn* ^*ΔΧBs6*^ [37], may also participate in recognition.

The identification of and orientation toward congeners is flexible and variable. Plasticity of identification and navigation toward congeners while avoiding aliens may be a means by which larvae cope with and adapt to changing and essentially unpredictable environments. The presence of larvae of two or more species within a decaying fruit unit and, consequently, of alien larval odors, is difficult to predict. We conjecture that the presence/absence of alien larval volatiles in a fruit unit is a circumstance that promotes flexibility and plasticity in the navigation of *Drosophila* larvae. More specifically, the environmental uncertainty created by female egg-laying site selection could act as an evolutionary pressure on the organization and function of the nervous system of *Drosophila* larva.

### Olfaction is not required for navigation in larvae of a natural population of *D*. *melanogaster*

A key finding in this study is the apparent absence of olfactory perception for navigation in larvae of the Trana isolate (Figs 1 **D**, 1**L**, 2**D** and 2**L**). These findings contrast with the behavior of the Til-Til isolate larvae of the same species (Figs 1**C**, 1**K**, 2**C** and 2**K**). The Trana strain was obtained from adults that emerged from grapes of the País variety. Only *D*. *melanogaster* adult flies emerged from these grapes. The decay process in grapes of the País variety produces relatively high ethanol, acetaldehyde and acetic acid concentrations, and acidity may reach pH 3.0 (unpublished data). *D*. *melanogaster* larvae are exceptional for their ability to utilize and tolerate high concentrations of these substances, whereas larvae of other species exhibit less or no tolerance [38]. By contrast, from the decaying semitropical prickly pear fruits, at pH 6.31, the ancestors of the Til-Til strain of *D*. *melanogaster* emerged together with *D*. *simulans*, *D*. *buzzatii* and a few *D*. *pavani*. Thus, Til-Til larvae developed with larvae of other species of *Drosophila*. The deprivation of odor experience may lead to a loss of sensitivity and acuity for distinguishing odorants [39]. However, we cannot exclude the possibility that the Til-Til and Trana isolates also differ in the frequency of certain genes involved in the processing of olfactory cues [40, 41].

The above conjectures cannot be completely correct. In the presence of conspecific and *D*. *pavani* larval odors, pupae of the Trana strain were preferentially observed near the conspecific paper [6]. Taken together, these contrasting findings suggest that *D*. *melanogaster* larvae emit a variety of chemically different volatiles. Each odor could have a different function. Some of these aromas might be necessary to identify conspecifics and aliens at a distance. Other chemically different odor cues may be required to select pupation sites. Independent neurological circuits may be involved in processing these different larva odor inputs at a central level. Larvae of different natural populations of *D*. *melanogaster* may also exhibit quantitative differences in emission rates of these scents.

### Navigation of learning mutant larvae of *D*. *melanogaster*

Our data show that the *Syn* ^*97CS*^ and *rut* learning mutant larvae are unable to identify congeners and aliens (Figs 1**F–**1**H,** 1**N–**1**P**, 2**F–**2**H** and 2**N–**2**P**). These findings suggest social larval odor-based learning in identification and orientation of the larvae toward congeners. Additionally, the results for the *rut* mutation indicate that the neuronal circuits that control the identification of congeners and aliens are located at the larva brain. Wild type larvae of *D*. *simulans* and *D*. *buzzatii* reared in isolation away from their congeners pupated randomly across the substrate. These larvae form pupa aggregations when reared with congeners [14]. Larvae of *Drosophila hydei*, subgenus *Drosophila*, *repleta* group, *hydei* subgroup, and *Drosophila busckii*, subgenus *Dorsilopha*, also form pupa aggregations away from *D*. *melanogaster* and *D*. *pavani* pupae [14, 36]. These findings suggest that social larval odor-based learning seem to be widely distributed in *Drosophila* genus having an essential role in the identification of congeners and aliens. Future experiments should be focused on the navigation of wild type *D*. *melanogaster* larvae reared in isolation from congeners.

### The sibling species *D*. *pavani* and *D*. *gaucha*

The sibling species *D*. *pavani* and *D*. *gaucha* exhibited notable differences in olfactory sensorial system function in larvae (Figs 3 and 4), confirming and extending the results reported by Del Pino et al. [6]. In nature, Chilean populations of *D*. *pavani* and *D*. *melanogaster* coexist in the same orchards, whereas the Buenos Aires strain of *D*. *gaucha* is allopatric with respect to *D*. *pavani* and its larvae are ecologically isolated from those of *D*. *melanogaster* (unpublished data). This isolation from other species produces delays in processing and transmitting information to central structures [39]. We have not examined whether *D*. *gaucha* larvae respond to odor cues emitted by *D*. *pavani* larvae. In addition, adults of *D*. *simulans* and *D*. *buzzatii* may emerge from the same decaying cladodes of prickly pear used by *D*. *gaucha* as breeding sites. For definitive conclusions, *D*. *gaucha* larvae must be tested against larval volatiles of each of those three species.

### *D*. *pavani* x *D*. *gaucha* hybrid larvae

The divergent results for the *pavani* x *gaucha* F_1_ reciprocal hybrids confirm that the two sibling parental species differ remarkably in olfactory sensitivity for the identification of larvae of the same and other *Drosophila* species (Figs 3 and 4). The results suggest that the X chromosome of each of the two parental species include loci for the identification of congener and alien larvae, which also affect the orientation and navigation of the larvae. These loci may be linked with those involved in the selection of pupation sites [6]. The genes seem to exhibit behavioral pleiotropy for navigation across the substrates and selection of pupation sites.

Alternative alleles appear to be linked on the X chromosome of each species. However, these reciprocal differences in behavior in crosses between species might also reflex cytoplasmic heredity. Relatively little attention has been paid to olfactory responses implicated in identification and spatial orientation in inter- and intraspecific hybrid larvae of *Drosophila*. Studies of this type may provide valuable information about neurogenetics and the evolution of behavior. Definitive proof will require a comparative cellular and molecular analysis of chemosensory receptors and brain circuits in larvae of *D*. *pavani* and *D*. *gaucha* and the reciprocal hybrids.

### Locomotion

Locomotion reflects the functional state of the nervous system [42]. Thoracic and abdominal neuronal circuits control freely moving larvae [43]. Our data revealed no differences in the locomotion rate of larvae that freely move on agar (controls) and those moving in the presence of synthetic food aroma and of conspecific and alien larval odors (Table 1 and S1 Table). We conclude that the conspecific and alien larval odors studied principally affect larval brain circuit performance.

## Concluding Remarks

We have not chemically identified the nature of the molecules emitted by *Drosophila* larvae and recognized by their olfactory receptors. However, it is clear that these emissions perform an essential ecological function related to the partition and allocation of space and habitat selection and, presumably, food preferences.

The mechanism by which the absence of larvae of other species blocks the identification of congeners and aliens remains unclear. Combinations of behavior, electrophysiology, and genetic approaches should make it feasible to better decipher the role of the olfactory cues emitted by *Drosophila* larvae in those behaviors.

We propose that *Drosophila* larvae emit a number of scents, each related to a different ecological function. The production of these aromas could be regulated by the presence/absence of larvae of other *Drosophila* species, suggesting the crucial importance of female egg-laying sites selection for larval behavior. Very little is known about *Drosophila* larva pheromones [44]. On the other hand, recognition of congeners in *Drosophila* larva keeps them in proximity to individuals of their own species, which may also have consequences for later adult life and mating. Studies on *Drosophila* larva routines in the wild will be decisive in validating these hypotheses. In summary, *Drosophila* larvae exhibit sophisticated odor-based behaviors linked with the use of the space available within breeding sites.

## Supporting Information

S1 Fig Decaying apples (*Pyrus malus*, pink lady variety) showing bacterium and fungi communities consumed by *Drosophila* larvae. *D*. *simulans* and *D*. *melanogaster* adults emerged from the fruits.
(JPG)

S2 Fig Decaying oranges (*Citrus sinensis*, sequoia variety) showing bacterium and fungi communities. Larvae of *D*. *immigrans*, *D*. *busckii* and *D*. *melanogaster* were observed eating the microorganisms.
(JPG)

S1 Table The *t*-values yielded to compare locomotion of larvae of the same strain in four different experimental conditions (values in Table 1). (DOC)
