# Supplementary figures and images for: The Identification of Congeners and Aliens by Drosophila Larvae
Source: PLoS One. 2015 Aug 27;10(8):e0136363. doi: 10.1371/journal.pone.0136363 (PMC4552012; doi:10.1371/journal.pone.0136363)

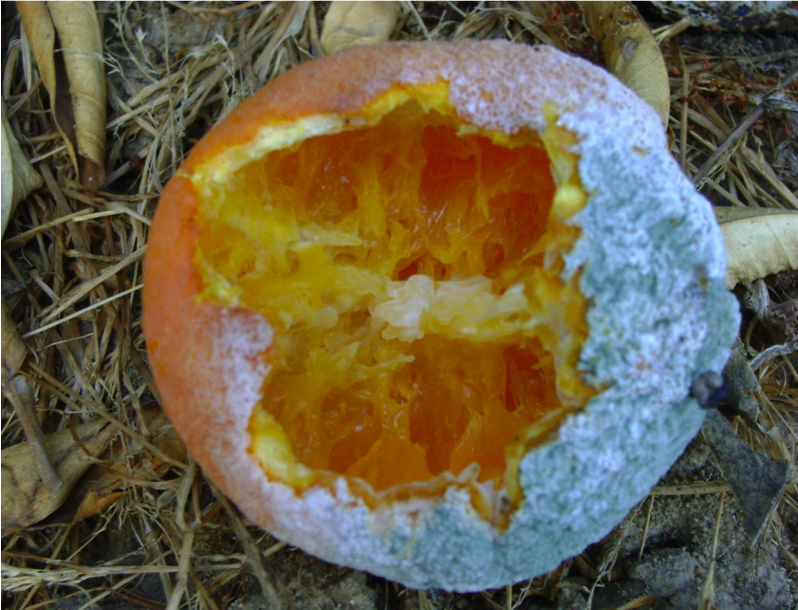

Supplement: S1 Fig — D. simulans and D. melanogaster adults emerged from the fruits. (JPG) [file pone.0136363.s001.jpg]

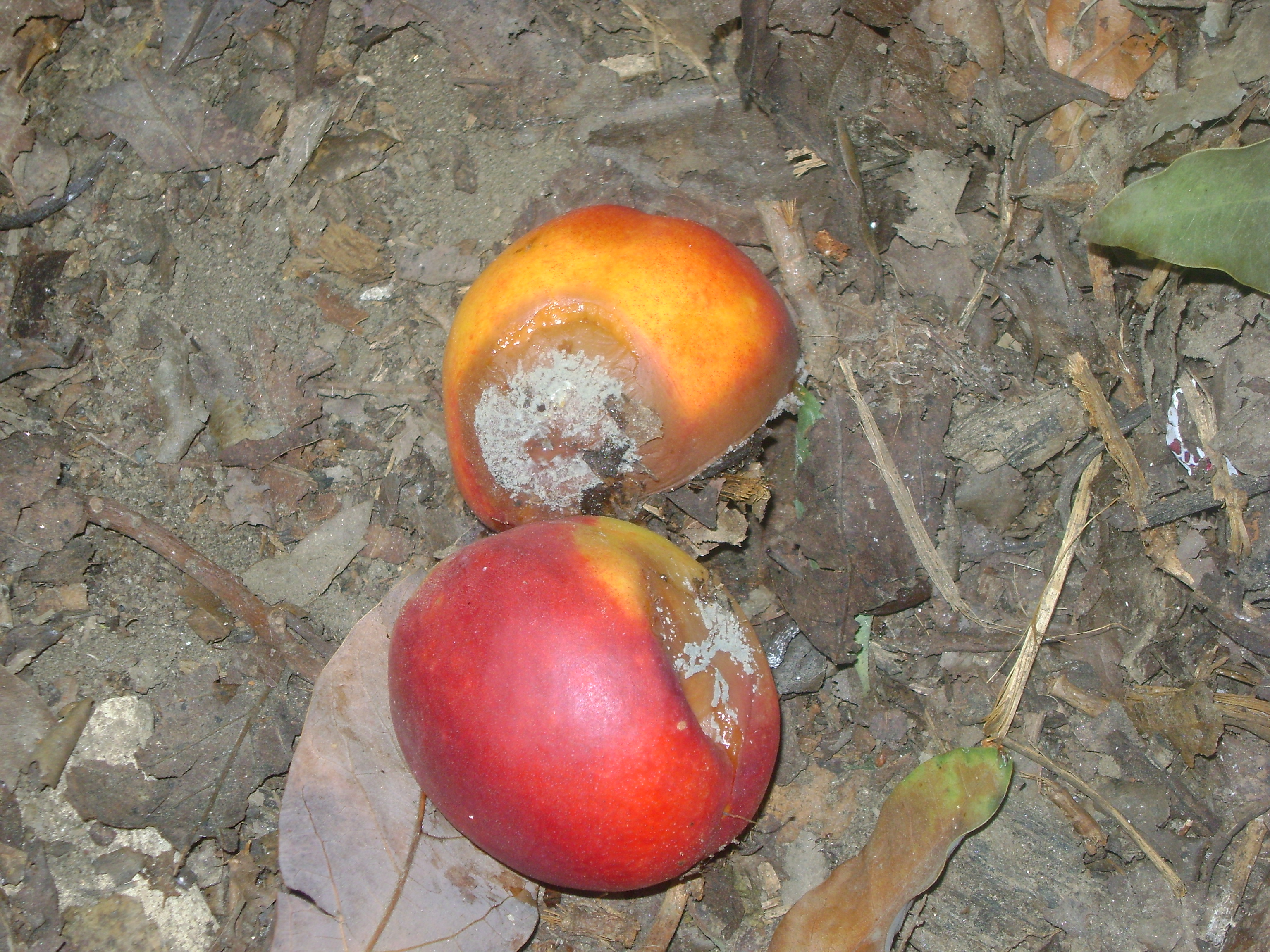

Supplement: S2 Fig — Larvae of D. immigrans, D. busckii and D. melanogaster were observed eating the microorganisms. (JPG) [file pone.0136363.s002.JPG]
