# Supplementary material for: The Identification of Congeners and Aliens by Drosophila Larvae
Source: PLoS One. 2015 Aug 27;10(8):e0136363. doi: 10.1371/journal.pone.0136363 (PMC4552012; doi:10.1371/journal.pone.0136363)
Supplement: S1 Table — (DOC) [file pone.0136363.s003.doc]

S1 Table. The *t*-values for differences in locomotion between larvae moving on agar and larvae of the same strain moving on agar plus odors emanated from sterile food, conspecific food and alien food. Locomotion rates are in Table 1 (see also Materials and Methods).

| **Species and strains** | ***t*-values for differences in locomotion on agar versus** | | |
| --- | --- | --- | --- |
|  | **agar + sterile food** | **agar + conspecific food** | **agar + alien food** |
| ***D. melanogaster*** |  |  |  |
| Oregon R-c | 1.06 | 1.05 | 0.95 |
| Canton-S | -0.01 | -0.47 | -0.19 |
| Til-Til | 0.09 | 0.12 | -0.20 |
| Trana | 0.27 | -0.38 | -0.16 |
| *vestigial* | 0.18 | 0.43 | 0.30 |
| *Orco* | -0.28 | -0.37 | -0.12 |
| *Syn^97CS^* | 1.09 | 1.46 | 0.06 |
| *rut* | -0.03 | 0.17 | -0.14 |
|  |  |  |  |
| ***D. pavani*** | -0.25 | -0.60 | -0.57 |
| ***D. gaucha*** | -0.06 | 0.11 | 0.87 |
|  |  |  |  |
| **The F1 hybrids** |  |  |  |
| *pavani* female x  *gaucha* male | 0.32 | -0.63 | 0.29 |
| *gaucha* female x  *pavani* male | -0.23 | -0.11 | 0.24 |

*t*-value_(0.05, 98)_ critical value = 1.984
